# Bactericidal action of positive and negative ions in air

**DOI:** 10.1186/1471-2180-7-32

**Published:** 2007-04-17

**Authors:** Louise A Fletcher, Lindsey F Gaunt, Clive B Beggs, Simon J Shepherd, P Andrew Sleigh, Catherine J Noakes, Kevin G Kerr

**Affiliations:** 1 School of Civil Engineering, University of Leeds, Leeds, LS2 9JT, UK; 2 Department of Electronics and Computer Science, University of Southampton, Southampton, SO17 1BJ, UK; 3 School of Engineering, Design and Technology, University of Bradford, Bradford, BD7 1DP, UK; 4 Harrogate Health Care Trust, Harrogate District Hospital, Lancaster Park Road, Harrogate HG2 7SX, UK

## Abstract

**Background:**

In recent years there has been renewed interest in the use of air ionisers to control of the spread of airborne infection. One characteristic of air ions which has been widely reported is their apparent biocidal action. However, whilst the body of evidence suggests a biocidal effect in the presence of air ions the physical and biological mechanisms involved remain unclear. In particular, it is not clear which of several possible mechanisms of electrical origin (i.e. the action of the ions, the production of ozone, or the action of the electric field) are responsible for cell death. A study was therefore undertaken to clarify this issue and to determine the physical mechanisms associated with microbial cell death.

**Results:**

In the study seven bacterial species (*Staphylococcus aureus*, *Mycobacterium parafortuitum*, *Pseudomonas aeruginosa*, *Acinetobacter baumanii*, *Burkholderia cenocepacia*, *Bacillus subtilis *and *Serratia marcescens*) were exposed to both positive and negative ions in the presence of air. In order to distinguish between effects arising from: (i) the action of the air ions; (ii) the action of the electric field, and (iii) the action of ozone, two interventions were made. The first intervention involved placing a thin mica sheet between the ionisation source and the bacteria, directly over the agar plates. This intervention, while leaving the electric field unaltered, prevented the air ions from reaching the microbial samples. In addition, the mica plate prevented ozone produced from reaching the bacteria. The second intervention involved placing an earthed wire mesh directly above the agar plates. This prevented both the electric field and the air ions from impacting on the bacteria, while allowing any ozone present to reach the agar plate. With the exception of *Mycobacterium parafortuitum*, the principal cause of cell death amongst the bacteria studied was exposure to ozone, with electroporation playing a secondary role. However in the case of *Mycobacterium parafortuitum*, electroporation resulting from exposure to the electric field appears to have been the principal cause of cell inactivation.

**Conclusion:**

The results of the study suggest that the bactericidal action attributed to negative air ions by previous researchers may have been overestimated.

## Background

In recent years there has been renewed interest in the use of air ionisers to control the spread of airborne infection. In particular, results from a clinical trial in an intensive care unit suggest that negative air ions may have the potential to control some hospital acquired infections (HAIs) [1]. One characteristic of 'air' ions which has been widely reported is their apparent biocidal action. Over many years, various researchers have reported that 'air' ions inhibit the growth of *Penicillium notatum *[2,3], *Neurospora crassa *[3], *Serratia marcescens *[4], "*Staphylococcus albus*" [5], *Candida albicans *[6], *Escherichia coli *[7], *Pseudomonas veronii *[8], *Aspergillus versicolor *[9], *Enterococcus malodoratus *[9], *Staphylococcus chromogenes *[9] and *Sarcina flava *[9]. However, while this body of work collectively suggests a biocidal effect in the presence of ions, in both air [2-7,9] and nitrogen [7,8], the physical and biological mechanisms involved remain unclear. In particular, it is not clear which of several possible mechanisms of electrical origin (i.e. the action of the ions, the production of ozone, or the action of the electric field) are responsible for cell death. A study was therefore undertaken to clarify this issue and to determine the physical mechanisms associated with microbial cell death. In this study seven bacterial species were exposed to both positive and negative ions in the presence of air for various durations and the bactericidal effects recorded.

## Results

**Figure 1:**
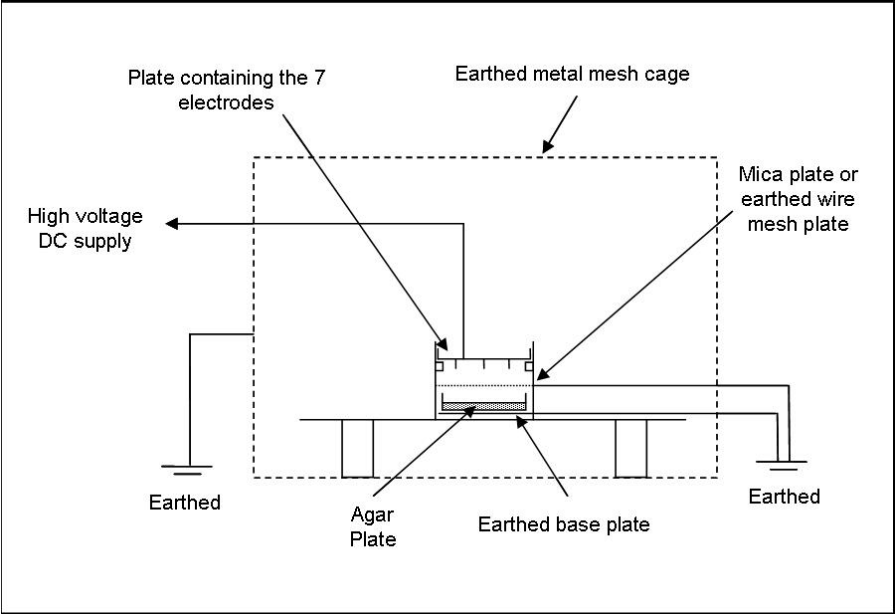
Diagram of the test rig.

In the study bacteria in pure culture on agar were exposed to air ions in a test rig, as shown in Figure 1. Experimentation was undertaken on sessile cultures of *Staphylococcus aureus *(NCTC 10399/ATCC 13709), *Mycobacterium parafortuitum *(NCTC 10410/ATCC 19687), *Pseudomonas aeruginosa *(NCIMB 10848), *Acinetobacter baumanii *(NCTC 12156/ATCC 19606), *Burkholderia cenocepacia *(NCTC 10744/ATCC25609), *Bacillus subtilis *(NCTC 10106/ATCC33234) and *Serratia marcescens *(NCTC 1377/ATCC 274. Cultures were exposed to positive air ions and negative air ions in separate experiments. In order to ensure uniform ionic exposure the agar plates were earthed and placed directly below a 7-pin electrode set, with the surface of the agar being 25 mm below the electrode tips. A high voltage generator (Brandenburg Alpha III, Brandenberg, UK) was used to apply a DC potential between the pin electrodes and the earthed agar plate. During experimentation an ammeter located on the earth conductor measured the current associated with the ions impinging on the agar plate.

In order to distinguish between effects arising from: (i) the action of the air ions; (ii) the action of the electric field, and (iii) the action of any ozone produced as a by-product of the corona discharge, two interventions were made to the experimental procedure. The first intervention involved placing a thin mica sheet between the 7-pin electrode set and the agar plate, which while leaving the electric field largely unaltered, prevented the air ions from reaching the bacteria. It should be noted that with free charges, the mica plate may have altered the space charge generated by the corona and thus may have modified the field distribution slightly. In addition, the mica plate prevented ozone generated by the electrical discharge from reaching the bacteria. The second intervention involved placing an earthed wire mesh 20 mm below the electrode set, and above the agar plates. This had the effect of greatly reducing (i.e. to negligible levels) the electric field strength and the number of air ions to which the bacteria were exposed. However, ozone generated by the discharge could still reach the agar plate.

All the ion exposure experiments were undertaken under ambient room conditions. All seven bacterial species were exposed to negative air ions (with an electrode potential of -10 kV) for periods of 5, 10 and 15 minutes, with five replicates taken on each occasion. The process was then repeated for samples exposed to positive ions, with an electrode potential of +10 kV. The negative ion exposure experiments were repeated twice more, first with a mica sheet placed above the agar plate and then with an earthed wire mesh located as described above. During experimentation the earth current and moisture loss from the agar plates were recorded. Ozone levels were also monitored using a portable detector (Model A-21ZX, Eco Sensors, USA).

**Figure 2:**
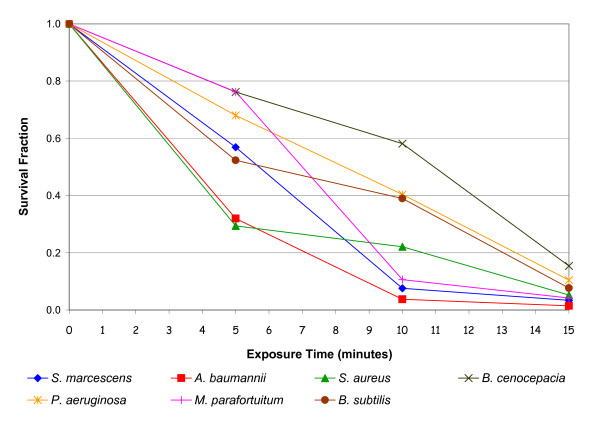
Reduction in viability of bacteria following negative energisation of the electrodes. The survival fractions shown are the mean of 5 replicates.

The results of the negative air ion experiment (i.e. without any interventions) are presented in Figure 2, which shows the survival fraction verses exposure time for each bacterial species. From these it can be seen that in every case exposure to negative air ions was associated with a marked reduction in colony count. Statistical analysis using a T-test (two-tailed with equal variance) reveals that for all bacteria species this reduction was significant (*p *< 0.01). Interestingly, little difference was observed between the behaviour of the Gram-positive and Gram-negative species.

**Figure 3:**
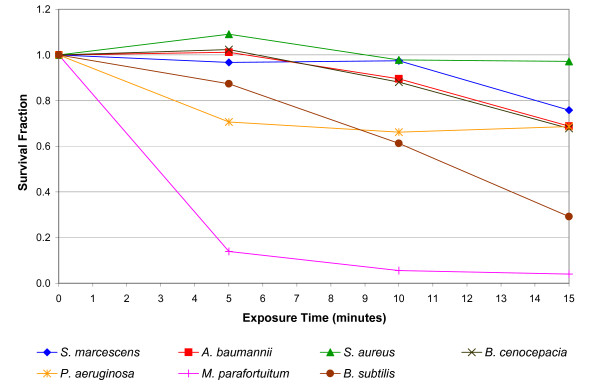
Reduction in viability of bacteria following positive energisation of the electrodes. The survival fractions shown are the mean of 5 replicates.

The results of the positive air ion experiment are presented in Figure 3. In marked contrast to the negative ion results, it can be seen that exposure to positive ions produced a dissimilar effect in the various test bacteria. A substantial reduction was observed for *Mycobacterium parafortuitum *(96.0% after 15 minutes exposure) (*p *< 0.001) and *Bacillus subtilis *(70.8% after 15 minutes) (*p *< 0.001), with a much smaller reduction for *Pseudomonas aeruginosa *(31.4% after 15 minutes) (*p *< 0.001), *Acinetobacter baumanii *(31.2% after 15 minutes) (*p *< 0.001), *Burkholderia cenocepacia *(32.1% after 15 minutes) (*p *= 0.059) and *Serratia marcescens *(24.2% after 15 minutes) (*p *= 0.002). However, no bactericidal effect was observed for *Staphylococcus aureus *(*p *= 0.345). With the exception of *Mycobacterium parafortuitum *the bactericidal effect produced when the bacteria were exposed to positive ions was much less than that achieved by the negative ions.

**Figure 4:**
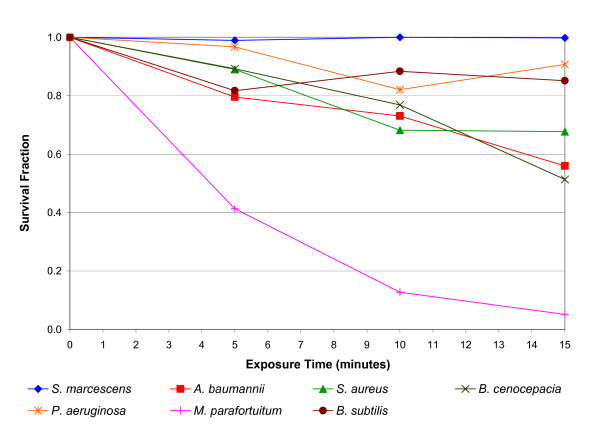
Reduction in viability of bacteria following negative energisation of the electrodes, with the intervention of a mica plate. The survival fractions shown are the mean of 5 replicates.

The results of the mica plate experiment, revealing the effect of the electric field alone, are presented in Figure 4. Comparison of these results with those in Figure 2 reveals that the intervention of the mica plate had a marked effect on the bactericidal action of the negative ions. This suggests that for most of the bacteria species tested, the bactericidal action observed in Figure 2 was not primarily due to the action of the electric field. However, from Figure 4 it can be seen that for one species in particular, *Mycobacterium parafortuitum*, the action of the electric field alone appears to have resulted in a strong bactericidal effect, with a 94.9% reduction (*p *< 0.001) occurring after 15 minutes exposure. Lesser reductions were also observed for *Burkholderia cenocepacia *(48.7% after 15 minutes) (*p *< 0.001), *Acinetobacter baumanii *(44.0% after 15 minutes) (*p *< 0.001), *Staphylococcus aureus *(32.3% after 15 minutes) (*p *= 0.005), with only marginal reductions occurring for *Bacillus subtilis *(14.9% after 15 minutes) (*p *= 0.516) and *Pseudomonas aeruginosa *(9.3% after 15 minutes) (*p *= 0.192).

**Figure 5:**
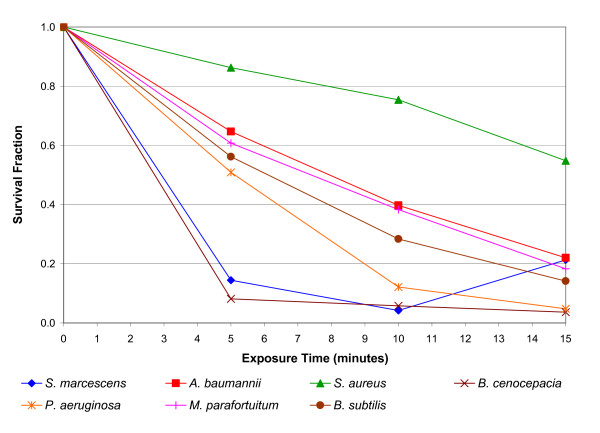
Reduction in viability of bacteria following negative energisation of the electrodes, with the intervention of a wire mesh. The survival fractions shown are the mean of 5 replicates.

The results of the wire mesh experiment, in which the action of the electric field and the air ions were reduced to negligible levels, are presented in Figure 5. All the bacterial species exhibited marked reductions (*p *< 0.01) in colony count during the exposure period. This indicates that all the bacteria were susceptible to ozone damage.

The mean currents (together with the maximum and minimum values) recorded during the various experiments are presented in Table 1. These values for the positive and negative ion experiments (i.e. columns 2 and 3) indicate that although the potential of the electrode was constant for the each experiment, there was some variation in the current produced. This phenomenon was probably due to variations in the conductivity of the agar, and day-to-day variations in temperature, humidity and air movement in the laboratory. With regard to the mica plate experiment, it can be seen that the current flow was negligible, indicating the lack of any ion flow to the agar plate. Similarly, the earthed wire mesh prevented any current flowing to the agar plate.

During production of negative ions the ozone concentration in the vicinity of the agar plate was found to be 2.3 ppm. However, during positive ion production this value was found to be considerably lower, about 0.8 ppm.

## Discussion

Although a number researchers have noted the phenomenon of microbial death 'when exposed to air ions', to the authors' best knowledge, none of the previous studies have made a rigorous attempt to separate out the various effects of electrical origin that may be responsible for cell death. For example, Kellogg *et al*. [5] and Digel *et al*. [9] made no attempt to distinguish effects arising from ozone or electric fields, from those caused by air ions themselves. By contrast Shargawi *et al*. [6] and Noyce and Hughes [7,8] did attempt to eliminate the effects of ozone, by performing a series of negative 'air' ion experiments in a pure nitrogen atmosphere. However this approach is inherently flawed, because although negative air ions readily form in air, they cannot form in pure nitrogen, and the mechanism is therefore believed to be electronic. One feature of negative coronas is that they can only be sustained in fluids which contain electronegative molecules, such as O_2_, H_2_O and CO_2_, since these gases have molecules which readily scavenge free electrons. Without electronegative molecules to capture free electrons, small negative cluster ions cannot form, with the result that a simple flow of electrons will occur in an ionized gas between the two electrodes and an arc will develop. Therefore any direct comparison between the action of negative ions in air and in nitrogen, such as that performed by Shargawi *et al*. [6] is somewhat erroneous, since both ozone and negative ions will form in air but not in nitrogen. Furthermore, irrespective of the nature of the atmosphere used, an electric field will always be present and this may have an impact on microbial viability. Given this, it is difficult to draw any firm conclusions about possible bactericidal mechanisms from much of the previous work.

Experiments such as described in this paper and those of previous researchers can produce microbial death by three, quite distinct, electrical phenomena:

• **Electrodynamics **– Ions, electrons and other ionising radiations

• **Electrostatics **– Electric charge/Electric field

• **Electro-chemical effects **– Ozone production

The experiments described in this paper have been designed to distinguish, for the first time, between these different effects. A mica plate was used to isolate the microorganisms from the ions and any ozone produced by the electric discharge. Similarly, an earthed wire mesh was used to prevent exposure of the microorganisms to both the ions and the electric field, allowing only the ozone to pass. In this way, it was possible to quantify with some accuracy the relative proportions of microbial mortality attributable to the different bactericidal mechanisms.

From the data presented in figures 2, 4 and 5 a clear consistent picture emerges. When exposed to 'negative ions' the principal bactericidal mechanism affecting most of the test species appears to be oxidation damage arising from exposure to ozone. This is clearly evident from Figure 5 which shows the reduction in the microbial population primarily due to the action of ozone. This finding echoes that of Shargawi *et al*. [6], who when working with *Candida albicans *found a strong correlation between cell death and the level of ozone present. The results achieved with the mica plate in place reinforce the opinion that ozone played an important role in the inactivation of most of the species tested. The results in Figure 4 indicate that for most of the species tested the negative ions and the electric field played only a minor role in cell death compared with the action of the ozone. Further evidence supporting this comes from the positive ion data presented in Figure 3. The mechanism by which positive ions are produced generates much less ozone than does its negative counterpart. This is because of the greater number of free electrons associated with negative coronas and the fact that the reactions which produce ozone are relatively low-energy. Consequently, when exposed to positive ions the test bacteria were also exposed to reduced levels of ozone compared with levels associated with negative air ionization, with the result that for most of the test species only a modest bactericidal action was observed. Interestingly, from the results in figures 2 and 5 it appears that some microorganisms (i.e. *Serratia marcescens *and *Acinetobacter baumannii*) are more susceptible to ozone alone compared with the combined action of ozone, negative ions and the electric field. However, the reasons for this phenomenon are unclear.

The behaviour of *Mycobacterium parafortuitum *is of particular interest. For this bacterium in the absence of ozone or negative ions (see Figure 4), a substantial reduction (i.e. 94.9% at 15 minutes) was achieved solely through the intervention of the electric field. This suggests that unlike the other bacteria, with this species the principal inactivation mechanism is electroporation. Corroboration of this comes from Figure 5 which shows that for this bacterium, the kill achieved through the action of ozone alone was actually less than that achieved by the electric field alone. Further evidence suggesting that ozone played only a relatively minor role in the inactivation of *Mycobacterium parafortuitum *comes from Figure 3, where it can be seen that under conditions of reduced ozone a large kill was still achieved. Of the bacteria tested this behavior appears unique to *Mycobacterium parafortuitum*. By comparison, the electric field appears to have played only a minor role in the inactivation of the other bacteria.

Collectively the data in figures 2, 4 and 5 suggests that for most of the bacterial species tested, negative air ions play only a relatively minor role in the bactericidal process. Microbial inactivation appears to occur mainly through a combination of ozone damage and damage caused by the electric field, with very little contribution from the negative ions themselves. Although not conclusive, Figure 3 suggests that the positive ions also have only a very limited affect on the bacteria studied. Indeed, the similarity between figures 3 and 4 is striking. Thus, a very interesting picture emerges. It would appear that some bactericidal action results from all the electrical phenomena tested. The disinfectant properties of ozone are well known and this mechanism seems to be responsible for the majority of the kills. However, it also appears that both the electric field and air ions have a contributory role.

Although it might be argued that the magnitude of the applied field is not high in relative terms, the complete dynamics of the time development of the electric field in a spherical dielectric shell representing the cellular membrane can be obtained using an analytical solution of the Ohmic conduction problem. Indeed, it has been found that the field in the membrane can reach a maximum value two orders of magnitude higher than the original Laplacian electrical field [10]. The plasma membrane of a cell serves the vital function of partitioning the contents of the cytoplasm from the external environment. These membranes are largely composed of amphiphilic lipids which self-assemble into highly insulating structures and thus present a large energy barrier to trans-membrane ionic transport. However, the lipid matrix can be disrupted by a strong external electric field leading to an increase in trans-membrane conductivity and diffusive permeability. These effects are the result of formation of aqueous pores in the membrane, which alter the electrical potential across the membrane and may ultimately lead to cell lysis.

Electroporation is a mechanical method used to introduce polar molecules into a host cell through the cell membrane. In this procedure, a large electric pulse temporarily disturbs the phospholipid bilayer, allowing molecules like DNA to pass into the cell. Electroporation is also the basic mechanism of tissue injury in high-voltage electric shock. If the strength of electrical field and duration of exposure to it are properly chosen, the pores formed by the electrical pulse reseal after a short period of time, during which the extracellular compounds have a chance to get inside the cell. However, excessive exposure of live cells to electrical fields can also cause apoptosis. Indeed, such harsh treatments have been used for killing tumour cells.

Care has been taken in the experimental protocols described in this paper to distinguish between the potential biocidal effects of the ions and the possibility of cell death induced by the electric field alone. Onset of cell lysis generally takes place at electric field strengths of around 100 V/mm or equivalently 100 kV/m. The ionising electrodes in our experiments are located 25 mm from the agar surface. At a potential of 10 kV this results in an 'estimated' field strength of 400 kV/m, which suggests that cell death due to electroporation may indeed have occurred. (It should be noted that because of the large field gradient at the electrode tip, this simple computation over-estimates the field strength at the agar surface. However, for the purposes of this study this estimate is a useful enough indicator of the field strength at the agar surface.) To quantify the effect of the electric field, a mica barrier was inserted between the ion source and the agar surface. The mica stopped the ions and ozone but had little effect on the electric field. In this way it was possible to measure the background effect of the electric field in isolation. From Figure 4 it can be seen that in addition to the dramatic effect on *Mycobacterium parafortuitum*, the electric field alone appears to have resulted in a modest kill in most of the other microbial species tested.

Collectively, the findings of the study indicate that negative air ions have little bactericidal effect on the species studied, despite being generated in close proximity to the microorganisms. This confirms the observations of Kerr *et al*. [1], who in a study on an intensive care unit (ICU), found the action of negative air ions to be associated with; (a) increased environmental isolates of *Acinetobacter *spp, and (b) a marked decrease in numbers of *Acinetobacter *infections/colonizations. Clearly, if the negative ions had had any bactericidal effect, then Kerr *et al*. would have observed a decrease in environmental isolates. This suggests that in the ICU study the reduction in observed *Acinetobacter *infections/colonizations was mainly due to physical effects rather than any bactericidal phenomena.

## Conclusion

The results presented in this paper suggest that the bactericidal action attributed to negative air ions by previous researchers may have been overestimated. With the exception of *Mycobacterium parafortuitum*, the principal cause of cell death amongst the bacteria studied was exposure to ozone, with electroporation playing a secondary role. However in the case of *Mycobacterium parafortuitum*, electroporation resulting from exposure to the electric field appears to have been the principle cause of cell inactivation. Further work is needed to determine whether this a feature common to all *Mycobacterium *species or indeed other bacteria with a similar cell wall structure such as *Nocardia *spp.

## Methods

The bacterial strains used in the study were stored at -20°C prior to use and were then used to aseptically inoculate 100 ml of tryptone soya broth (TSB). The TSB was incubated in a shaking incubator for 24 hours at 37°C (48 hours in the case of *M. parafortuitum*). The concentration of each culture was determined by serial dilution followed by inoculation of 0.1 ml onto tryptone soya agar plates. Following incubation at 37°C for 24 or 48 hours the number of colonies was counted and the concentration of bacteria in the original culture was determined. Based on this information appropriate dilutions of the cultures were carried out to give a concentration of approximately 3000 cfu/ml. Sterile tryptone soya agar (TSA) plates were subsequently inoculated with 0.1 ml of this dilution, which was spread evenly over the surface of the media with a sterile spreader, to yield approximately 300 colonies per plate. The plates were then allowed to dry at room temperature before being used in the ion exposure experiments. For each exposure period five identical replicate plates were prepared.

Following ion exposure the agar plates were incubated at 37°C for a period of 24 or 48 hours after which time the number of colonies on each plate was counted. Control plates were treated in an identical manner with the exception of exposure to the ion source.

## Authors' contributions

LAF carried out the experimental work and prepared microbiological samples. LFG and SJS designed the test rig and participated in the design of the study. PAS, CJN and KGK participated in the design of the study. CBB conceived and coordinated to the study, drafted the manuscript and undertook the statistical analysis. All authors have read and approved the manuscript.

## Figures and Tables

**Table 1:** Mean currents recorded during experiments, with range indicated in brackets.

**Bacterium**	**Average Current (range) (μA)**			
	
	Negative ions	Positive ions	Negative ions with mica plate	Negative ions with wire mesh
*Serratia marcescens*	76.0 (65 – 95)	42.3 (30 – 50)	0.000 (0 – 0.005)	0.001 (0 – 0.01)
*Acinetobacter baumanii*	102.0 (40 – 150)	35.3 (30 – 45)	0.003 (0 – 0.005)	0.000 (0 – 0)
*Staphylococcus aureus*	65.00 (30 – 90)	36.0 (25 – 45)	0.003 (0 – 0.05)	0.000 (0 – 0)
*Burkholderia cenocepacia*	109.0 (95 – 120)	32.0 (30 – 35)	0.022 (0 – 0.2)	0.000 (0 – 0)
*Pseudomonas aeruginosa*	97.0 (80 – 105)	36.3 (30 – 40)	0.003 (0 – 0.025)	0.000 (0 – 0)
*Mycobacterium parafortuitum*	113.0 (95 – 130)	36.3 (25 – 45)	0.009 (0 – 0.05)	0.000 (0 – 0)
*Bacillus subtilis*	114.3 (90 – 135)	27 (20 – 40)	0.002 (0 – 0.025)	0.000 (0 – 0)
